# EVALUATING THE IMPACT OF THE ALZHEIMER’S ASSOCIATION HELPLINE

**DOI:** 10.1093/geroni/igae098.1901

**Published:** 2024-12-31

**Authors:** Nancy Hodgson, Sonia Talwar, Liming Huang, Kerry Finegan, Sam Fazio

**Affiliations:** University of Pennsylvania, Philadelphia, Pennsylvania, United States; University of Pennsylvania, Philadelphia, Pennsylvania, United States; University of Pennsylvania, Philadelphia, Pennsylvania, United States; Alzheimer’s Association, Chicago, Illinois, United States; Alzheimer’s Association, Chicago, Illinois, United States

## Abstract

Most persons living with dementia in the US live at home and are supported by family. The Alzheimer’s Association national Helpline offers free 24/7 support, including telephone care consultations. Three iterative studies evaluating the Alzheimer’s Association Helpline were conducted between January 2019 and December 2023. The first study compared the benefit of the Helpline Care Consultation to a Care Consultation Plus program on the emotional and mental health of 445 callers (75% White, 80% women). The second study compared outcomes in 1822 callers (75% White, 79% women) randomly assigned to staff who provided the traditional care consultation model, or those trained in a brief solution-focused approach. The third study (2503 callers – 80% women, 73% White) evaluated the efficacy of up to two additional care consultations. In all three studies, we observed statistically significant improvement in self-efficacy in managing emotions. In the first study, callers’ mental health and self-efficacy improved significantly. In the second study callers assigned to the solution-focused approach had higher therapeutic alliance than callers assigned to the traditional care consultation. The self-efficacy scores in the third study were significantly lower in callers that requested an additional. Self-efficacy improved significantly in those who opted for one or two calls. Support services provided via the Helpline can be very effective at improving caregiver self-efficacy, managing emotions and ability to take action. Additional research is indicated to understand the diverse needs of caregivers and what types of supportive services work best for Helpline callers.

